# hUCMSCs carrying exenatide prevent T1DM by improving intestinal microflora composition and islet tissue damage repair

**DOI:** 10.1186/s10020-022-00526-0

**Published:** 2022-12-13

**Authors:** Wei Wang, Yahao Wang, Jingwei Chi, Xiaojun Tan, Jianxia Hu, Xiaolong Ma, Xiaofang Sun, Kui Che, Wenshan Lv, Yangang Wang

**Affiliations:** 1 Department of Hematology, The Affiliated Hospital of Qingdao University, Qingdao, 266000 People’s Republic of China; 2 Medical College, Qingdao University, Qingdao, 266071 People’s Republic of China; 3 Key Laboratory of Thyroid Diseases, Medical Research Center, The Affiliated Hospital of Qingdao University, Qingdao, 266000 People’s Republic of China; 4 Department of Endocrinology, Yidu Central Hospital of Weifang City, Weifang, 261000 People’s Republic of China; 5 The Laboratory of Thyroid Disease, The Affiliated Hospital of Qingdao University, Qingdao, 266000 People’s Republic of China; 6 Department of Endocrinology, Liaocheng People’s Hospital, Liaocheng, 252000 People’s Republic of China; 7 Department of Endocrinology, The Affiliated Hospital of Qingdao University, No. 16, Jiangsu Road, South District, Qingdao, 266000 Shandong People’s Republic of China

**Keywords:** Exenatide, Human umbilical cord mesenchymal stem cells, Type 1 diabetes mellitus, Intestinal flora abundance, Islet tissue damage

## Abstract

**Background:**

Exenatide is a stable analogue of glucagon-like peptide 1 that can reduce postprandial hyperglycemia and has been utilized as adjunctive therapy for type 1 diabetes mellitus (T1DM). The human umbilical cord is a rich source of MSCs, and human umbilical cord mesenchymal stem cells (hUCMSCs) also show potential to enhance insulin secretion. Here, we aimed to explore the effects of hUCMSCs carrying exenatide in T1DM and further identify the possible mechanisms involved.

**Methods:**

hUCMSCs were isolated from human umbilical cord tissues, identified, and transduced with recombinant lentivirus carrying exenatide to obtain exenatide-carrying hUCMSCs (hUCMSCs@Ex-4).

**Results:**

The results showed that hUCMSCs@Ex-4 restored the blood glucose levels and body weight of NOD mice, and repressed immune cell infiltration and islet tissue changes. Additionally, in T1DM mice, treatment with hUCMSCs@Ex-4 reduced the blood glucose levels and promoted repair of islet tissue damage. Moreover, hUCMSCs@Ex-4 attenuated renal tissue lesions in T1DM mice. Applying bioinformatic analysis, the effects of hUCMSCs@Ex-4 were suggested to correlate with decreased abundance of pro-inflammatory intestinal bacteria and increased abundance of anti-inflammatory intestinal bacteria.

**Conclusion:**

Overall, the study indicated that hUCMSCs carrying exenatide might improve beneficial intestinal microflora abundance and promote islet tissue damage repair, thereby alleviating T1DM.

**Supplementary Information:**

The online version contains supplementary material available at 10.1186/s10020-022-00526-0.

## Introduction

Type 1 diabetes mellitus (T1DM) is identified as a chronic autoimmune disease marked by selective autoimmune-regulated damage of pancreatic islet beta-cells, which can cause an absolute lack of insulin (Gregory et al. 2013). T1DM is often diagnosed at young age (Li et al. 2017), and this lifelong disease usually presents as abnormally increased blood glucose levels, chiefly due to insulin dysfunction (Yang et al. 2019). T1DM is a complex disease, and the risk factors mainly include genetic susceptibility, immune dysregulation and environmental exposures (Acharjee et al. 2013). Of note, the gut microbiome plays a significant role in the pathogenesis of T1DM (Zheng et al. 2018). Unfortunately, despite the improved treatment of T1DM by use of exogenous insulin, the majority of patients fail to meet clinical glycaemic goals (Dayan et al. 2019). Recently, mesenchymal stem cells (MSCs) have been suggested as a potential tool for the therapy of T1DM (Bohacova and Holan 2018).

The umbilical cord is considered a rich source of MSCs that can be harvested to treat refractory diseases through allogeneic cell therapy (Tipnis et al. 2010). Umbilical cord mesenchymal stem cells (UCMSCs) are understood to be distinctive, available, and a non-disputable source of early stem cells, which are also easy to apply (Yang et al. 2012). As previously reported, human UCMSCs (hUCMSCs) can contribute to both in vitro and in vivo secretion of insulin, thereby demonstrating promise for treating T1DM (Boroujeni and Aleyasin 2014). In addition, islet-like clusters derived from MSCs in the Wharton's Jelly of the human umbilical cord are found capable of converting into insulin-producing cells that may improve T1DM, with a series of advantages in terms of availability and underlying risks (Chao et al. 2008). UCMSCs’ potential towards pancreatic/islet lineage has led to their consideration as a cell therapy product for future human islet transplantation programs to treat diabetes (Kadam et al. 2012). Exenatide, a type of synthetic exendin-4, is a stable analogue of glucagon-like peptide 1, which has been approved for the clinical use against type 2 diabetes mellitus (Nathanson et al. 2009). Notably, adjunctive exenatide therapy has been shown to decline postprandial hyperglycemia in adolescents with T1DM even in the presence of reduced insulin dose; thus, exenatide bears significant therapeutic potential for adjunctive treatment of T1DM (Raman et al. 2010). Adipose-derived stem cells carrying exendin-4 have been demonstrated to alleviate diabetic wound healing (Seo et al. 2017). Similarly, MSCs with exendin-4 are shown to induce improvements in mitochondrial dysfunction in diabetic cardiomyopathy (Wassef et al. 2018). However, the role of hUCMSCs carrying exenatide has not been explored in the context of T1DM.

Through high-throughput sequencing, dysbiosis of intestinal microflora has been unveiled to be related to intestinal inflammation and loss of microbial short chain fatty acids (SCFAs), which are two potential pathogenic factors implicated in the pathogenesis of T1DM (Ma et al. 2020). Treatment with human MSCs is shown to reverse gut microbiome dysbiosis, whereby suppressing inflammation and hepatic steatosis in the setting of non-alcoholic steatohepatitis (Yang et al. 2020). Antibiotics-induced depletion of intestinal microflora is seen to reverse hyperglycemia in diabetic mice to potentiate the efficacy of MSC therapy (Lv et al. 2020). The possible effects of hUCMSCs carrying exenatide on intestinal microflora composition in the context of T1DM are yet unknown. Therefore, in this study, we set out to investigate whether exenatide-carrying hUCMSCs can improve intestinal microflora composition and islet autoimmunity in T1DM.

## Materials and methods

### Ethical approval

The study protocol was approved by the Ethics Committee of The Affiliated Hospital of Qingdao University. All clinical study procedures were in accordance with the *Declaration of Helsinki.* All participants signed informed consent documents before sample collection. Great efforts were made to minimize the suffering of the experimental animals.

### Culture of hUCMSCs

Fresh human umbilical cords were collected from 6 parturients (aged 23–38 years) at The Affiliated Hospital of Qingdao University from March 2016 to May 2017. Each umbilical cord sample was cut into 1–2 mm^3^ pieces and suspended in Dulbecco’s modified Eagle’s medium (DMEM)/F12 (1:1; L310KJ; Shanghai Basalmedia Technologies Co., Ltd., Shanghai, China) containing 10% fetal bovine serum (FBS; Biowest, Nuaillé, France), 100 U/mL penicillin, and streptomycin in a 5% CO_2_ incubator at 37 °C. The medium was changed every 3 days.

Upon reaching 80–90% confluence, the cells were trypsinized and transferred to a new petri dish for further expansion. The cell phenotype was analyzed by flow cytometry and a hUCMSC phenotype was identified (Additional file 1: Fig. S1A, B). The cells were trypsinized and washed twice with PBS solution, and a single cell suspension was blocked with 2% rabbit serum (Stemcell Technologies, Vancouver, BC, Canada) at room temperature for 30 min. The cells were then labeled with fluorescent anti-human antibodies (1:200; Thermo Fisher Scientific, Inc., Waltham, MA, USA) against CD73-fluorescein isothiocyanate (FITC; 11-0739-41), CD90-FITC (11-0909-41), CD105-phycoerythrin (PE; 12-1057-41), CD34-FITC (11-0349-41), CD45-FITC (11-0459-41), CD14-FITC (11-0149-42), CD19-PE (50-102-58), and HLA-DR-FITC (11–9952-41) at 4 °C for 30 min. The cells were washed thrice with PBS, resuspended in PBS (1 mM EDTA), and analyzed on a FACSCalibur™ flow cytometer (BD Bioscience, Franklin Lakes, NJ, USA). All data were analyzed using FlowJo (version 8.7; Tree Star, Inc., Ashland, OR, USA).

### Animal treatments

Recombinant lentivirus carrying exenatide was prepared. In short, eukaryotic signal peptide (SP) sequence was selected by prediction software. A DNA fragment was cloned into the 5'end of GFP of the pCMV-EGFP vector and transduced into 293T cells. Western blot analysis was used to determine the content of GFP in the culture medium to identify the SP sequence with the highest extracellular transport efficiency. The best SP sequence was then linked to the 5'end of exenatide cDNA and cloned into pCMV-MCS vector. Lentivirus was prepared by using plasmid packaging system. Preparation of hUCMSCs carrying exenatide (hUCMSCs@Ex-4): the recombinant lentivirus carrying exenatide was transduced into hUCMSCs and the infection rate was estimated to be about 98% based on fluorescence labeling in the viral vector.

A total of 12 healthy female C57BL/6 mice and 60 female non-obese diabetic (NOD) mice (Beijing Vital River Laboratory Animal Technology Co., Ltd., Beijing, China) were housed individually in a specific-pathogen-free laboratory at 22–25 °C with 60–65% humidity under a 12 h light/dark cycle, with ad libitum access to food and water for one week before the experiment. The health of the mice was verified before the experiment. C57BL/6 mice were injected with 0.3 mL PBS via the tail vein and served as control. NOD mice were randomly assigned to groups and separately treated with PBS (injected with 0.3 mL PBS via tail vein), hUCMSCs@Ex-4 (injected with hUCMSCs carrying 2 μg/kg exenatide [Baxter, Dearfield, Illinois, USA] via tail vein), insulin (treated with the same volume of insulin glargine), exenatide (injected with 2 μg/kg exenatide via tail vein), or hUCMSCs (injected with 0.3 mL PBS containing 1 × 10^6^ hUCMSCs via tail vein) (n = 12 for each). During the experiment, the body weight and blood glucose levels of mice in different treatment groups were recorded weekly.

For validating the localization of hUCMSCs in the pancreas, hUCMSCs were labeled with chloromethyl-benzamidodialkylcarbocyanine (CM-Dil; Life Technologies, Eugene, Oregon, USA) according to the manufacturer’s instructions, and then injected into the mice via tail vein (Yin et al. 2018). Five days after the injection, the mice were euthanized and the pancreas were excised and prepared into frozen sections for observation.

In order to further study whether hUCMSCs@Ex-4 could treat T1DM, a T1DM mouse model was constructed. Healthy and clean female NOD mice aged 6–8 weeks were selected and acclimated for one week. These mice were fed a normal diet with free access to water during this week (Ding et al. 2020). Acclimation was necessary to induce T1DM. The health status of the animals should be checked before the experiment; mice with neat, smooth, and shiny hair, as well as clean ears, nose, and mouth, were regarded as healthy mice. From the second week, the mice were weighed at 2:00 p.m. once a week, and blood was collected by acupuncture at the tail. Blood glucose was monitored using a ONETOUCH Ultra blood glucose meter (Johnson & Johnson, New Brunswick, NJ, USA). T1DM could be diagnosed with two consecutive random blood glucose values ≥ 16.6 mmol/L.

Twelve healthy female C57BL/6 mice and 72 T1DM mice were used. C57BL/6 mice were injected with 0.3 mL PBS via tail vein and served as control. The T1DM mice were treated with PBS (T1DM mice injected with 0.3 mL PBS via tail vein on the 3rd day after the onset of the disease); hUCMSCs@Ex-4 (T1DM mice injected with hUCMSCs carrying 2 μg/kg exenatide via tail vein on the 3rd day after the onset of the disease); insulin (T1DM mice treated with 0.3 mL insulin glargine on the^3rd^ day after the onset of the disease); exenatide (T1DM mice injected with 2 μg/kg exenatide via tail vein on the 3rd day after the onset of disease); hUCMSCs (T1DM mice injected with 1 × 10^6^ hUCMSCs via tail vein on the 3rd day after the onset of disease). These mice were followed-up, during which their general conditions were observed and recorded every day, including the amount of food, water, urine, and mental responses. The day when the mice were diagnosed with T1DM was recorded as the 1st day (0 week) and the body weight and random blood glucose levels were measured. From then onwards, the T1DM mice were weighed and the random blood glucose level was determined on the 2nd, 4th, 6th, 8th week as described above. Besides, the serum C peptide level of T1DM mice was determined after 16 h of fasting.

One week after the injection of hUCMSCs@Ex-4, an oral glucose tolerance test (OGTT) was carried out. The mice were given 1.5 g/kg glucose orally after starvation for 6 h, and blood glucose value was then measured at 0, 15th, 30th, 60th and 120th minutes.

During the entire experiment, the mice in each group responded to the drug treatment without obvious side effects.

### Hematoxylin and eosin (HE) and immunohistochemical staining

The pancreatic tissues of mice were collected and cut into 8–10 pieces, fixed overnight with 10% neutral formalin, embedded in paraffin, sectioned and stained with HE. The paraffin-embedded pancreatic tissue sections were heated at 60 °C for 20 min, cleared twice in xylene for 15 min each, rehydrated in descending alcohol series, and treated with 3% H_2_O_2_ at room temperature for 10 min. Antigen retrieval was performed in sodium buffer, the sections were blocked with normal goat serum (Sangon Biotech Co., Ltd., Shanghai, China) at room temperature for 20 min, and probed overnight at 4 °C with diluted primary antibodies to insulin (ab181547, 1:500, Abcam), glucagon (G2654, 1:500, Sigma-Aldrich), CD4 (ab183685, 1:200, Abcam), CD8 (ab217344, 1:200, Abcam), CD11b (ab133357, 1:200, Abcam), CD11c (ab52632, 1:200, Abcam), Ki67 (ab15580, 1:300, Abcam), and WT-1 (ab89901, 1:300, Abcam). The next day, the sections were re-probed with secondary antibody goat anti-rabbit IgG (ab6721, 1:500, Abcam) or goat anti-mouse IgG (ab197774, 1:500, Abcam) for 30 min, treated with streptavidin biotin peroxidase complex (SABC; Vector Labs, Burlingame, CA, USA) and developed with DAB (Sigma-Aldrich). The sections were stained with hematoxylin for 30 s, dehydrated in ascending series of alcohol, cleared and mounted for observation under an upright microscope (BX63, Olympus Japan Co., Ltd., Tokyo, Japan). An image analysis system (Aperio Scanscope System, Vista, CA, USA) was utilized to determine the insulin-positive area. The cell area was calculated as the product of the total insulin-positive area multiplied by the corresponding pancreatic weight.

A Leica DMR microscope (Leica, Quebec, Canada) equipped with a Leica DC 300F camera was used for blinded examination of the islets to assess the degree of monocyte infiltration. The severity of the infiltration was categorized into 5 levels: (0) no infiltration/insulitis, (1) peripancreatic infiltration, (2) infiltration of the islet area less than 50%, (3) infiltration of the islet area greater than 50%, (4) The infiltration of the islet area was greater than 50% and it was insulin-negative, (5) pancreatic islet atrophy, with no stained insulin, no/few lymphocytes. The average insulitis score was calculated by dividing the sum of the insulitis scores by the total number of islets examined in each mouse. To calculate the mass of insulitis and β-cells, two sections were incised from each pancreas, at least 250 μm apart.

### Terminal deoxynucleotidyl transferase-mediated dUTP-biotin nick end labeling (TUNEL) assay

The pancreatic tissues of mice were embedded in paraffin, sectioned, heated at 60 °C for 20 min, cleared twice in xylene for 15 min each and rehydrated in descending series of alcohol. Next, TUNEL detection solution was prepared using the one-step TUNEL Apoptosis Detection Kit (C1088, Shanghai Beyotime Biotechnology Co., Ltd., Shanghai, China) as per the manufacturer’s instructions. The sections were washed twice with PBS, incubated with 50 μL TUNEL detection solution at 37 °C in the dark for 60 min, mounted, and observed under a fluorescence microscope (BX63, Olympus).

### Determination of biochemical index

Mice in each group were placed in independent metabolic cages, with free access to drink. The mice were fasted overnight before euthanasia, and 24 h urine samples were collected. All urine specimens are preserved with toluene. After 24 h, the urine volume was assessed. After centrifugation to remove the sediments, the samples were separately placed into 1.5 mL Eppendorf tubes, which were then stored in a freezer at -20 °C for subsequent use. The 24 h urine albumin excretion rate (UAER) was determined. Upon the end of the experiment, blood was collected by the inner canthal blood sampling method. After standing for 1 h, the blood samples were centrifuged at 3000 rpm for 10 min, and the supernatant was extracted and stored in an Eppendorf tube at − 20 °C. Finally, the levels of blood urea nitrogen (BUN) and serum creatinine (Scr) were determined using an automatic biochemical analyzer (RA-1000, Bayer, Leverkusen, Germany) with its supporting kits.

### Transmission electron microscope

After the mice of each group were euthanized, the kidney tissues were immediately removed and placed on an ice box to reduce cell autolysis. After removing the envelope, the renal cortex was cut into small pieces with a size of 1 × 1 × 1 mm, and fixed in 4% glutaraldehyde solution at 4 °C. The tissue pieces were then fixed using the acid-glutaraldehyde-osmium tetroxide fixation method, dehydrated in ascending series of alcohol, soaked in epoxy resin and embedded in porous rubber plates. Finally, the tissue pieces were sectioned, stained with uranium-lead, observed and photographed under a transmission electron microscope.

### Metagenomic shotgun sequencing

Fecal samples were selected from mice in the T1DM and hUCMSCs@Ex-4 groups, 12 samples per group. Genomic DNA (5 ng) per sample was extracted using the QIAamp Powerfecal DNA kit (QIAgen), and library preparation and subsequent whole genome sequencing (WGS) were performed using the Illumina HiSeq 2500 platform, with 150 bp pair-end read by 4 flow cytometry channels. The raw sequence data of the 24 samples contained an average of 31.5 million pairs of PE150 reads (with a standard deviation of 4.4 million) per sample. Filtering for quality was performed using Kneaddata v0.6.1 (https://huttenhower.sph.harvard.edu/kneaddata) with a sliding window of 3, a minimum quality score of 20, and a minimum sequence length of 100 bases. Both paired and orphan sequences that passed the initial filter were further purified using host (mus musculus) and PhiX (Illumina sequencing control) genomes. Then, quality-filtered reads from each sample were transmitted to HuMAnN2 v0.11.1 (https://github.com/biobakery/biobakery/wiki/humann2) for gene function and pathway analyses with default parameters. The abundance of a gene or pathway was re-normalized to relative abundance using the HuMAnN2 utility script. Data classification and effect analysis of the significantly differential species (ie LDA score) were carried out using the linear discriminant analysis effect size (LEfSe) method (https://github.com/biobakery/biobakery/wiki/lefse), with |LDA score|> 2 and *p* value < 0.05 as the criterion for significant difference.

### Enzyme-linked immunosorbent assay (ELISA)

Serum was obtained through centrifugation of the peripheral blood collected from the T1DM mice treated with hUCMSCs@Ex-4. ELISA kits were used to determine the levels of tumor necrosis factor α (TNF-α), interleukin (IL)-1β, IL-6, IL-10, IL-13 and lipopolysaccharide (LPS) in the serum of mice. The kits were purchased from eBioscience (Santiago, USA) and the levels of cytokines were determined based on the manufacturer’s instructions.

### Statistical analysis

Statistical analysis of data in this study was performed using SPSS 21.0 statistical software (SPSS, IBM, Armonk, NY, USA). Measurement data are expressed as mean ± standard deviation. Data from two groups were compared using unpaired *t*-test. Data comparisons between multiple groups at different time points were performed using Bonferroni-corrected repeated measures one-way analysis of variance (ANOVA). Spearman’s correlation analysis was performed to observe the correlation between indicators. *p* < 0.05 indicated that the difference was statistically significant.

## Results

### hUCMSCs@Ex-4 promoted the repair of islet tissue damage by reducing the blood glucose level of NOD mice

Human umbilical cord tissues were cultured for more than 15 days, and spindle-shaped fibroblasts were observed to grow and reach confluence (Additional file 1: Fig. S1A). Subsequently, hUCMSCs were isolated and extracted, and the phenotype of hUCMSCs was identified using flow cytometry. The results showed that CD73, CD90, and CD105 were positive while CD34 (hematopoietic stem cells), CD45 (white blood cells), CD14 (bone marrow cells), CD19 (B cells), and HLA-DR (DC, macrophages and B cells) were negative in the isolated cells (Additional file 1: Fig. S1B), indicating the successful isolation of hUCMSCs from umbilical cord tissues. In addition, CM-Dil-labeled hUCMSCs were detected in mouse pancreatic tissues (Additional file 1: Fig. S1C), which confirmed the localization of hUCMSCs in pancreatic tissues.

**Fig. 1:**
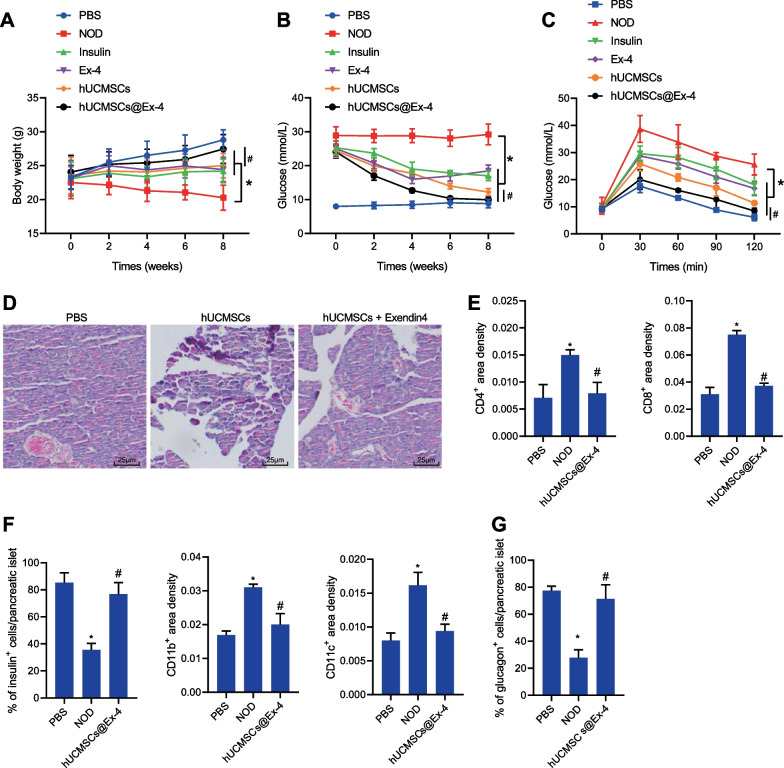
hUCMSCs@Ex-4 promotes the repair of islet tissue damage by reducing the blood glucose level in NOD mice. **A** Body weight of NOD mice after different treatments. **B** Blood glucose level in NOD mice with different treatments. **C** Blood glucose level in NOD mice with different treatments measured by OGTT. **D** HE staining for islet morphology in NOD mice with different treatments (400 ×). **E** Immunohistochemical staining analysis of the infiltration of immune cells (CD4^+^ T, CD8^+^ T, CD11b^+^, and CD11c^+^) in the pancreatic tissues of NOD mice with different treatments. **F** Proportion of β-cells in the pancreatic tissues of NOD mice with different treatments analyzed by immunohistochemical staining. **G** Proportion of α-cells in the pancreatic tissues of NOD mice with different treatments analyzed by immunohistochemical staining. In panels A-C, *p < 0.05 *vs.* NOD mice. ^#^*p* < 0.05, NOD mice treated with hUCMSCs@Ex-4 *vs.* NOD mice treated with hUCMSCs. In panels E–G, *p < 0.05 *vs.* PBS-treated mice. ^#^*p* < 0.05 *vs.* NOD mice. n = 12

Initially, we aimed to explore the therapeutic effect of hUCMSCs@Ex-4 in NOD mice. The results showed a decline in the blood glucose level with an increase in the body weight of mice treated with hUCMSCs, insulin, exenatide, or hUCMSCs@Ex-4 as compared to PBS-treated NOD mice. Importantly, mice treated by hUCMSCs@Ex-4 presented the most significant changes, with a lower blood glucose level and higher body weight relative to hUCMSCs treatment, showing statuses close to the PBS-treated mice (Fig. 1A, B). Moreover, mice in the different treatment groups were starved for 6 h and then orally given 1.5 g/kg glucose in the OGTT, and results indicated that the blood glucose levels in all the mice were elevated following oral administration of 1.5 g/kg glucose, which reached their peak at 30th minute and then decreased to the baseline blood glucose levels at 120^th^ minute after glucose administration. Relative to the PBS-treated NOD mice, the blood glucose level was lowered at 30^th^, 60^th^, 90^th^, and 120^th^ minutes in the mice treated with insulin, exenatide, hUCMSCs or hUCMSCs@Ex-4, with the lowest blood glucose levels induced by hUCMSCs@Ex-4; hUCMSCs@Ex-4 treatment exerted a more significant effect than hUCMSCs (Fig. 1C). In addition, HE staining data showed that the islet morphology of the pancreatic tissues of the PBS-treated mice was relatively intact, without lymphocyte infiltration. However, the islets of the mice treated with hUCMSCs@Ex-4 or the PBS-treated NOD mice exhibited different degrees of damage. In addition, lymphocyte infiltration around the islets was repressed by treatment with hUCMSCs@Ex-4 in NOD mice (Fig. 1D).

The immunohistochemical staining results of the immune cell markers (CD4 + T cells, CD8 + T cells, CD11b + cells, and CD11c + cells) suggested no inflammatory cell infiltration around the islet cells in the pancreatic tissues of the PBS-treated mice but mild inflammatory cell infiltration in the NOD mice after treatment with hUCMSCs@Ex-4. However, inflammatory cell infiltration was obvious around the islet cells in the pancreatic tissues of PBS-treated NOD mice (Fig. 1E). Insulin and glucagon immunohistochemical staining results revealed that the morphology of the islet β-cells and α-cells was intact in the PBS-treated mice while loss of islet β-cells and α-cells was observed in NOD mice. In the presence of hUCMSCs@Ex-4, the cell morphology was relatively clear and intact (Fig. 1F, G). In summary, these findings suggested hUCMSCs@Ex-4 could induce a beneficial therapeutic effect on NOD mice by reducing the blood glucose level and attenuating pancreatic tissue damage.

### hUCMSCs@Ex-4 reduced blood glucose levels to promote the repair of islet tissue damage in T1DM mice

**Fig. 2:**
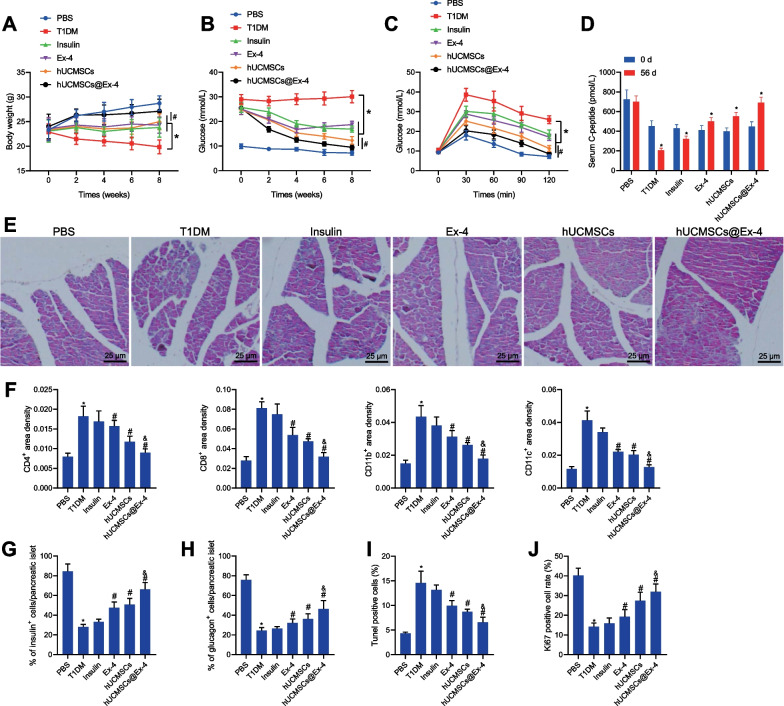
hUCMSCs@Ex-4 reduces blood glucose levels and promotes the repair of islet tissue damage in T1DM mice. **A** Curve showing body weight changes of T1MD mice with different treatments. **B** Curve for blood glucose changes of T1MD mice with different treatment. **C** Blood glucose level in T1MD mice with different treatments, determined by OGTT. D, Fasting serum C peptide levels in T1MD mice with different treatments, analyzed by ELISA. **E** HE staining of islet morphology in T1MD mice with different treatments (400 ×). **F** Immunohistochemical staining analysis of the infiltration of immune cells in the pancreatic tissues of T1MD mice with different treatments. **G** Proportion of β-cells in the pancreatic tissues of T1MD mice with different treatments, analyzed by immunohistochemical staining for insulin. **H** Proportion of α-cells in the pancreatic tissues of T1MD mice with different treatments, analyzed by immunohistochemical staining for glucagon. **I** TUNEL-positive islet cells in the pancreatic tissues of T1MD mice with different treatments. **J** Ki67 immunohistochemical staining of islet cells in the pancreatic tissues of T1MD mice with different treatments. In panels, *p < 0.05 *vs.* T1DM mice. ^#^*p* < 0.05 *vs.* T1DM mice treated with hUCMSCs@Ex-4 *vs.* T1DM mice treated with hUCMSCs. In **F**–**J**, *p < 0.05 *vs.* PBS-treated mice. ^#^*p* < 0.05 *vs.* T1DM mice. ^&^*p* < 0.05 *vs.* T1DM mice treated with hUCMSCs

Next, we proceeded to investigate whether hUCMSCs@Ex-4 can be used for the treatment of T1DM. As shown in Fig. 2A, B, the body weight of T1DM mice was decreased while the blood glucose was increased. The body weight was increased but the blood glucose was reduced in T1DM mice upon treatment with insulin, exenatide, hUCMSCs or hUCMSCs@Ex-4, among which hUCMSCs@Ex-4 induced more marked changes than hUCMSCs. Additional OGTT results exhibited significant elevations in the blood glucose levels of all mice upon oral glucose administration, which reached their peak at 30^th^ minute and decreased to the baseline blood glucose levels at 120^th^ minute after glucose administration. The blood glucose levels of T1DM mice were lowered at 30, 60, 90, and 120 min following treatment with insulin, exenatide, hUCMSCs or hUCMSCs@Ex-4, with the lowest blood glucose levels induced by hUCMSCs@Ex-4 treatment; hUCMSCs@Ex-4 treatment caused lower blood glucose levels versus hUCMSCs treatment (Fig. 2C).

Next, changes in fasting serum C peptide levels among T1DM mice were analyzed by ELISA. Compared with 0 week, the serum C-peptide level at 8 week was decreased in the T1DM mice treated with insulin or T1DM, while it was increased in T1DM mice treated with exenatide, hUCMSCs, or hUCMSCs@Ex-4, among which, treatment with hUCMSCs@Ex-4 contributed to more significant increases relative to hUCMSCs (Fig. 2D). HE staining demonstrated relatively intact islets in the pancreatic tissues of the PBS-treated mice without lymphocyte infiltration. In contrast, the T1DM mice displayed no intact islet structure in the pancreatic tissues, accompanied by infiltration of a large number of inflammatory cells (Fig. 2E). Mice treated with insulin also showed significant infiltration of inflammatory cells (Fig. 2E). However, the islets of mice treated with hUCMSCs, exenatide, or hUCMSCs@Ex-4 were relatively intact, presenting normal morphology and fewer inflammatory cells (Fig. 2E), with hUCMSCs@Ex-4 exhibiting the best treatment effect. Labeling of immune cell markers (CD4 + T cells, CD8 + T cells, CD11b + cells and CD11c + cells) as well as insulin and glucagon labeling showed no inflammatory cell infiltration around the islet cells with an intact structure of islet β-cells and α-cells in the pancreatic tissue of the PBS-treated mice, whereas, massive inflammatory cell infiltration occurred with the absence of islet β-cells and α-cells in the T1DM mice. Insulin treatment attenuated the inflammatory cell infiltration around the islet cells in the T1DM mice but the structure of islet β-cells and α-cells remained disrupted. Only a small number of inflammatory cells were seen infiltrated in the T1DM mice treated with exenatide, hUCMSCs or hUCMSCs@Ex-4, corresponding to clear cell structure noted in the pancreatic tissues. Notably, hUCMSCs@Ex-4 induced a superior suppressive effect on inflammatory cell infiltration and cell structural damage relative to either exenatide or hUCMSCs (Fig. 2F–H).

The results of TUNEL assay and Ki67 immunohistochemical staining demonstrated that islet cell apoptosis was reduced, while their proliferation was enhanced in the pancreatic tissues of the T1DM mice upon treatment with insulin, exenatide, hUCMSCs or hUCMSCs@Ex-4. Relative to the insulin-treated mice, a more pronounced reduction in islet cell apoptosis with relatively increased cell proliferation was seen in the pancreatic tissues of T1DM mice treated with exenatide, hUCMSCs, or hUCMSCs@Ex-4, of which hUCMSCs@Ex-4 resulted in the most obvious changes (Fig. 2I, J).

These results indicated that hUCMSCs@Ex-4 could reduce blood glucose levels and promote the repair of islet tissue damage in T1DM mice.

### hUCMSCs@Ex-4 alleviated the pathological changes in kidney tissues of T1DM mice

High glucose levels induced by diabetes can induce kidney damage, nephropathy, and even renal failure (Singh et al. 2018). The effect of hUCMSCs@Ex-4 on kidney tissue lesions in T1DM mice was, therefore, the subsequent focus of the study. As shown in Additional file 2: Table S1, the serum levels of BUN and Scr and 24 h UAER level were higher and the number of podocytes in kidney tissues was lower in T1DM mice as compared to PBS-treated mice. Following treatment with insulin, exenatide, hUCMSCs, or hUCMSCs@Ex-4, the above-mentioned changes in the T1DM mice were found reversed. Compared with insulin, exenatide led to mild increases in serum BUN and Scr levels and UAER level with reduced number of podocytes. In contrast, hUCMSCs or hUCMSCs@Ex-4 caused considerably lower serum BUN and Scr levels and UAER level with more podocytes, among which, hUCMSCs@Ex-4 led to the most evident changes. Also, hUCMSCs@Ex-4 led to increased serum BUN and Scr levels and UAER level but reduced reduced number of podocytes as compared to hUCMSCs.

**Fig. 3:**
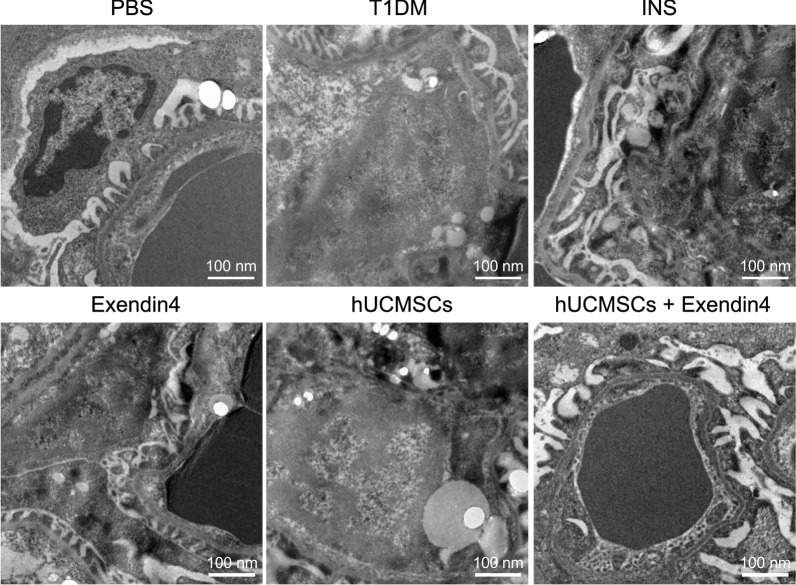
Changes in kidney tissues of mice with different treatments, observed under a transmission electron microscope (scale bar = 100 nm). n = 12

Under a transmission electron microscope, the glomerular capillary basement membrane in the T1DM mice was seen blurred and irregularly thickened compared to that of PBS-treated mice, and the podocytic process was damaged, fused, or even absent, accompanied by severe kidney tissue damage. Upon treatment with insulin, exenatide, hUCMSCs, or hUCMSCs@Ex-4, the pathological kidney tissue damage was significantly alleviated, with hUCMSCs@Ex-4 exerting a superior effect than hUCMSCs (Fig. 3). These results suggested that hUCMSCs@Ex-4 effectively alleviated the pathological changes in kidney tissues of T1DM mice.

### Bioinformatics analysis predicts that hUCMSCs@Ex-4 can promote the repair of islet tissue damage by reducing the abundance of pro-inflammatory bacteria in T1DM mice and enhancing the abundance of anti-inflammatory bacteria

**Fig. 4:**
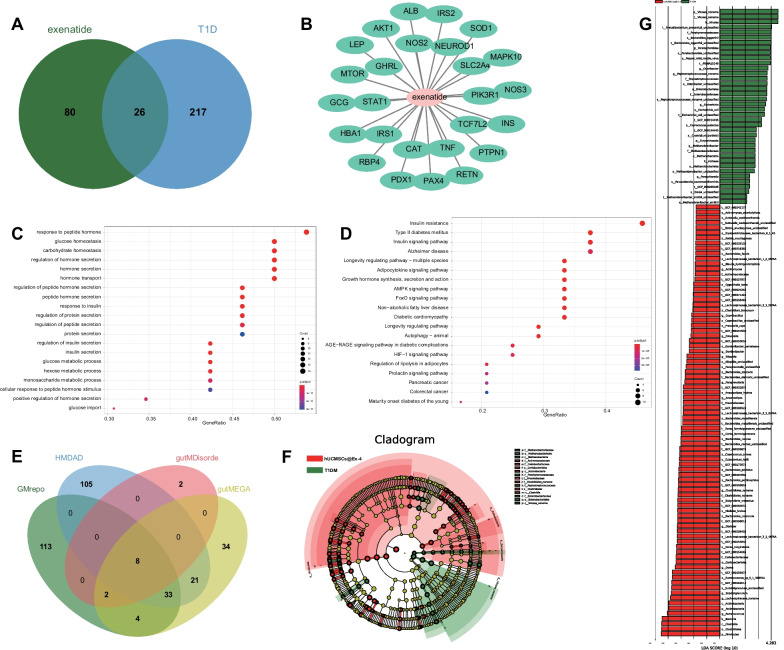
Bioinformatics analysis to screen the signaling pathways related to hUCMSCs@Ex-4 in the treatment of T1DM and changes in intestinal microflora. **A** Venn diagram of the exenatide-related genes and T1DM target genes determined using GeneCards. **B** The drug-target regulatory network. **C** GO enrichment analysis of 26 target genes. D, KEGG enrichment analysis of the 26 target genes. **E** Venn diagram of T1DM-related intestinal microflora predicted by the GMrepo, HMDAD, gutMEGA, and gutMDisorder databases. **F** Fecal intestinal microflora specie cladogram of T1DM mice treated with hUCMSCs@Ex-4 (red) and T1DM mice (green). **G** Differential fecal intestinal microflora species between T1DM mice treated with hUCMSCs@Ex-4 (red) and T1DM mice (green)

Using the database GeneCards we retrieved 106 target genes of exenatide and 243 T1DM-related genes (screening relevance score ≥50), and 26 target genes were obtained after intersecting the above genes (Fig. 4A). A drug-target regulatory network was obtained using Cytoscape 3.5.1 software (Fig. 4B). GO and KEGG enrichment analyses of the 26 target genes showed that the target genes of hUCMSCs for the treatment of T1DM were mainly enriched in signaling pathways such as insulin resistance, regulation of insulin secretion, etc. (Fig. 4C, D), associated with the function of regulating insulin secretion to repair pancreatic tissue damage. These results suggest that hUCMSCs@Ex-4 can promote the repair of pancreatic tissue damage and prevent the occurrence and development of T1DM.

The screening by GMrepo, HMDAD, gutMEGA, and gutMDisorder databases identified 160, 167, 12, and 102 relevant intestinal bacteria, respectively. In the intersection, seven significantly differentially expressed intestinal floras were obtained: *Escherichia*, *Haemophilus*, *Bacteroides*, *Prevotella*, *Eubacterium*, *Phascolarctobacterium*, and *Dialister* (Fig. 4E). The HMDAD database showed the changes of these intestinal floras in T1DM group versus the control group (Additional file 2: Table S2). Furthermore, we performed metagenomic sequencing on the feces of the T1DM mice and hUCMSCs@Ex-4-treated T1DM mice and analyzed the specie compositions and functional compositions of intestinal feces after quality control and host removal. The LEfSE differential analysis revealed that anti-inflammatory bacteria such as *Dialister*, *Phascolarctobacterium*, and *Prevotella* were dominantly presented in the T1DM mice treated with hUCMSCs@Ex-4, while the T1DM mice mainly presented with pro-inflammatory bacteria such as *Escherichia*, *Eubacterium*, and *Haemophilus* (Fig. 4F–G).

These results indicated dysbiosis of intestinal microflora in T1DM and increased pro-inflammatory bacteria. hUCMSCs@Ex-4 might reduce the abundance of pro-inflammatory bacteria and increase the abundance of anti-inflammatory bacteria, thereby promoting the repair of islet damage, and preventing the occurrence and development of T1DM.

### hUCMSCs@Ex-4 promote the secretion of anti-inflammatory cytokines and inhibit the secretion of pro-inflammatory cytokines in T1DM mice, significantly associated with the abundance of intestinal bacteria

**Fig. 5:**
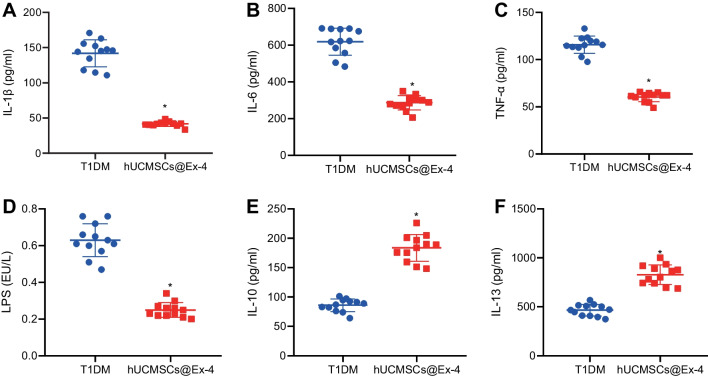
hUCMSCs@Ex-4 reduces the secretion pro-inflammatory cytokines while increasing that of anti-inflammatory cytokines in the serum of T1DM mice. **A** Serum levels of IL-1β in T1DM mice treated with hUCMSCs@Ex-4 measured by ELISA. **B** Serum levels of IL-6 in T1DM mice treated with hUCMSCs@Ex-4 measured by ELISA. **C** Serum levels of TNF-α in T1DM mice treated with hUCMSCs@Ex-4 measured by ELISA. **D** Serum levels of LPS in T1DM mice treated with hUCMSCs@Ex-4 measured by ELISA. **E** Serum levels of IL-10 in T1DM mice treated with hUCMSCs@Ex-4 measured by ELISA. F, Serum levels of IL-13 in T1DM mice treated with hUCMSCs@Ex-4 measured by ELISA. *p < 0.05 *vs.* T1DM mice. n = 12

ELISA results showed that the serum levels of pro-inflammatory cytokines (IL-1β, IL-6, TNF-α, and LPS) were significantly reduced in mice treated with hUCMSCs@Ex-4, while those of anti-inflammatory cytokines (IL-10 and IL-13) were markedly increased compared with the T1DM mice (Fig. 5).

In addition, the abundance change in *Prevotella* was positively correlated with the expression of IL-1β (*p* = 0.004); the abundance change in *Escherichia* was negatively correlated with the expression of IL-10 (*p* = 0.043) and IL-13 (*p* = 0.008); the abundance change in *Phascolarctobacterium* was negatively correlated with IL-1β (*p* = 0.014) and IL-6 (*p* = 0.014) and positively correlated with the expression of IL-13 (*p* = 0.003); the abundance change in *Bacteroidetes* presented negative correlations with IL-1β (*p* = 0.005), IL-6 (*p* = 0.006), TNF-α (*p* = 0.014), and LPS (*p* = 0.012) expression; the abundance change of *Haemophilus* showed positive correlations with IL-1β (*p* = 0.001) and LPS (*p* = 0.009) expression; the abundance change in *Prevotella* shared negative correlations with the expression of IL-1β (*p* = 0.040), IL-6 (*p* = 0.016), and TNF-α (*p* = 0.001) expression, and positive correlation with IL-10 (*p* = 0.040) and IL-13 (*p* = 0.011) expression; the abundance change in *Eubacterium* was positively correlated with the expression of IL-6 (*p* = 0.007) and TNF-α (*p* = 0.016), and was negatively correlated with IL-10 (*p* = 0.028) and IL-13 (*p* = 0.001) expression; the abundance change in *Dialister* was inversely correlated with IL-6 (*p* = 0.001) expression, and positively correlated with IL-10 (*p* = 0.044) and IL-13 (*p* = 0.037) expression (Additional file 2: Table S3). The aforementioned results indicated that hUCMSCs@Ex-4 could reduce the expression of pro-inflammatory cytokines in the serum of T1DM mice, and promote the expression of anti-inflammatory cytokines. The abundance of most anti-inflammatory intestinal bacteria shared a positive correlation with the expression of the anti-inflammatory cytokines, and the expression of pro-inflammatory intestinal bacteria was positively correlated with the expression of pro-inflammatory cytokines, ultimately contributing to the repair of islet tissue damage.

## Discussion

Stem cells carrying exenatide have been highlighted as an effective cell-based therapy for multiple diseases. Adipose-derived stem cells with exendin-4 can enhance bone repair (Deng et al. 2019). MSCs with exendin-4 have also shown significant alleviation of diabetic cardiomyopathy relative to the use of MSCs alone (Wassef et al. 2018). hUCMSCs are believed to be a valuable option for disease therapy (Chen et al. 2020). However, whether hUCMSCs@Ex-4 can be applied in the treatment of T1DM has not been addressed and was thus explored in this study.

We first showed that hUCMSCs@Ex-4 reduced the blood glucose level of NOD and TIDM mice. Previous research has highlighted the role of hUCMSCs and exenatide in regulating the blood glucose level in diabetes mellitus. A previous study revealed that differentiated insulin-producing cells from hUCMSCs resulted in alleviation of hyperglycemia (T1DM) in NOD mice (Wang et al. 2011). Moreover, hUCMSCs, when co-cultured with pancreatic cells, could be differentiated into islet-like cells to reduce blood glucose levels in a rat model of diabetes mellitus (Wang et al. 2014). Another study has suggested that exenatide exerts an inhibitory effect on the secretion of glucagon during either euglycemia or hyperglycemia but not affects glucagon and counter-regulatory hormones in the hypoglycemia setting in T1DM patients (Jiang et al. 2018). In addition, exenatide leads to marked declines in hemoglobin A1c levels and plasma glucose concentration in T1DM patients and does not increase hypoglycemia occurrence (Harris and Boland 2016). Partially concordant with these previous findings, the present study offered findings regarding the inhibitory impact of hUCMSCs and exenatide on the blood glucose level in NOD and TIDM mice, and further our study provided additional evidence that hUCMSCs@Ex-4 exerted superior glucose-lowering effect than hUCMSCs or Ex-4 alone.

An important finding of our study was that hUCMSCs@Ex-4 reduced the blood glucose levels in NOD and T1DM mice and further enhanced islet damage repair in T1DM mice. Specifically, the HE staining results showed that the extent of damage of islet morphology in the mice treated with hUCMSCs@Ex-4 was significantly less than that in the T1DM mice. Insulin and glucagon immunohistochemical staining suggested that treatment with hUCMSCs@Ex-4 in mice can significantly improve the morphological structure of pancreatic islet β-cells and α-cells, and reduce the infiltration of inflammatory cells compared with the T1DM mice. An positive immunomodulatory effect of exercise has been highlight in relation to the onset of T1DM and inflammation, but only moderate intensity training exerts the glucose-lowering role in the late diabetic stage (Codella et al. 2015). According to earlier reports, MCP-1 and IL-6 secreted from hUCMSCs can partially inhibit inflammation and induce polarization of M2 macrophages to restore islet function in type 2 diabetic mice (Yin et al. 2018). Therefore, we speculated that hUCMSCs might enhance the therapeutic effect of exenatide by downregulating inflammation in pancreatic islets. Specifically, UCMSCs are able to accumulate in damaged tissues and facilitate tissue repair while regulating immune response (Nagamura-Inoue and He 2014). In addition, UCMSCs can differentiate into functional islet cells, which can contribute to the release of physiological insulin as well as C-peptide, thereby holding promise as a therapeutic agent for the treatment of diabetes (Sarang and Viswanathan 2016). Additionally, UCMSCs derived from Wharton's jelly can repair the function of islet β-cells and are thus proposed as potential therapy for T1DM (Hu et al. 2013). In agreement with our findings, treatment with exenatide has been shown to result in marked improvement in islet function, in part by diminishing the levels of inflammation-related molecules in human pancreatic islets (Cechin et al. 2012). Moreover, exenatide may contribute to restoration of activity of normal genes in dysfunctional islets in normal and diabetic mice (Ghanaat-Pour and Sjoholm 2009). Park et. al. have revealed that exenatide can rescue impaired pro-islet amyloid polypeptide processing in cultured human islets, in part by inhibiting islet β-cell apoptosis and improving islet β-cell function (Park et al. 2013). Together with our results, it can be concluded that hUCMSCs@Ex-4 bear potential for beneficial islet function regulation in diabetes mellitus.

Furthermore, we identified seven distinct intestinal microfloras (*Escherichia*, *Haemophilus*, *Bacteroides*, *Prevotella*, *Eubacterium*, *Phascolarctobacterium* and *Dialister*) that were significantly associated with T1DM and demonstrated that hUCMSCs@Ex-4 improved intestinal microflora composition in NOD and T1DM mice. The species and functions of human intestinal microbiome in T1DM patients have shown disparity from healthy individuals (Ho et al. 2019; Dedrick et al. 2020). It has been illustrated that inflammatory response and loss of SCFAs in the intestine correlate with the intestinal microflora disorder, which is a critical mechanism in the pathogenesis of T1DM (Ma et al. 2020). Herein, pro-inflammatory microflora *Escherichia*, *Eubacterium*, and *Haemophilus* were enriched in the fecal samples of T1DM mice, revealing the potential significance of pro-inflammatory microflora in the pathogenesis of T1DM. A number of studies have further demonstrated the beneficial effect of hUCMSCs on intestinal microbiota in a variety of diseases. hUCMSCs were found to facilitate the re-establishment of the intestinal microbiota in food allergy induced by ovalbumin (Yan et al. 2018). Additionally, hUCMSCs were reported to exert protection against experimental colitis through CD5 + B regulatory cells (Chao et al. 2016). These findings made it plausible that hUCMSCs might function in the onset and development of T1DM through mediating intestinal microbiota-related inflammation. However, up to now, no evidence has linked exenatide to the imbalance of intestinal microbiota. In this study, LEfSE analysis suggested the enrichment of anti-inflammatory bacteria such as *Dialister*, *Phascolarctobacterium*, and *Prevotella* in the T1DM mice treated with hUCMSCs@Ex-4. This finding was consistent with subsequent evidence that hUCMSCs@Ex-4 treatment led to increased release of anti-inflammatory factors and reduced secretion of pro-inflammatory factors. Hence, it is r reasonable to conclude that hUCMSCs@Ex-4 probably contributes to the transformation of pro-inflammatory into anti-inflammatory intestinal microflora in T1DM, thus ameliorating islet tissue damage in T1DM.

## Conclusion

**Fig. 6:**
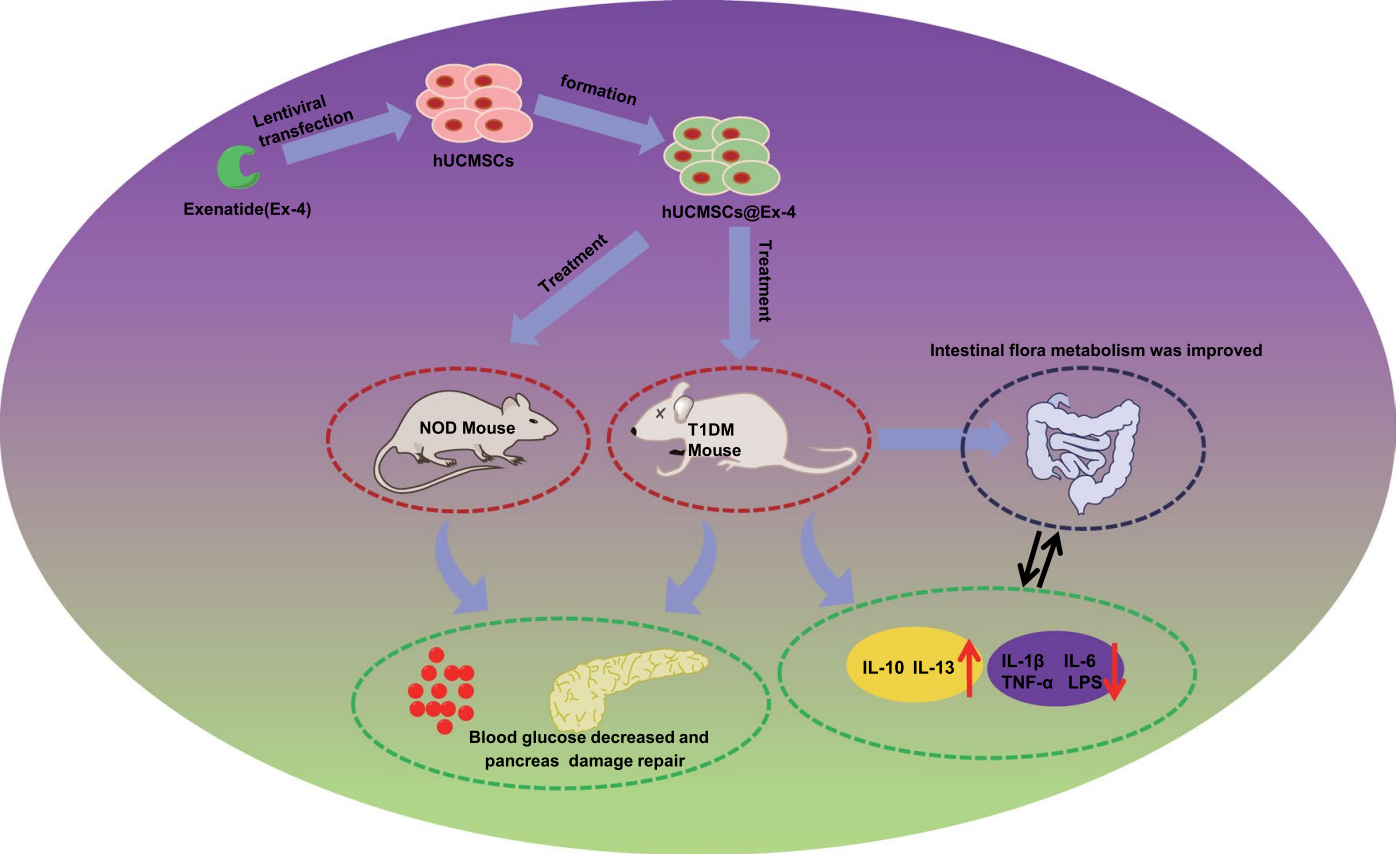
Schematic representation of the regulatory network mechanisms of hUCMSCs@Ex-4 effects in T1DM. hUCMSCs@Ex-4 reduces the secretion of pro-inflammatory cytokines and modulates the abundances of pro-inflammatory intestinal bacteria, and promotes the secretion of anti-inflammatory cytokines, increasing the abundances of anti-inflammatory intestinal bacteria, thereby reducing blood glucose levels and promoting pancreatic islet tissue damage repair, ultimately alleviating of T1DM

In conclusion, this study indicated that hUCMSCs@Ex-4 can potentially improve intestinal microflora composition and promote islet tissue damage repair in T1DM, thus alleviating T1DM (Fig. 6). These findings provide a novel direction for the development of cell-based therapy for T1DM. Of course, the potential mechanism by which hUCMSCs@Ex-4 could inhibit the occurrence and development of T1DM by regulating the intestinal flora is extremely complex. The results of our study could not fully reflect the real mechanism, and the conclusion of our study has certain limitations. In the future study, we will conduct more in-depth research and exploration.

## Supplementary Information

**Additional file 1: Fig. S1.** Identification of hUCMSCs. **A** Morphological images of umbilical cord tissues cultured for more than 15 days, seen under a Zeiss optical microscope (200 ×). **B** Flow cytometric analysis of CD34, CD45, CD73, CD90, CD105, CD14, CD19, and HLA-DR expression in hUCMSCs. **C** Localization of hUCMSCs in mouse pancreatic islets, observed under a fluorescence microscope (200×). Each experiment was run in triplicate independently.

**Additional file 2: Table S1.** The serum levels of BUN, Scr and UAER as well as the number of podocytes in mice with different treatments at the 8th week. **Table S2.** Changes in the differential intestinal floras in the control and T1DM groups in the HMDAD database. **Table S3**. Correlation analysis between serum inflammatory cytokines and intestinal floras in T1DM mice.

## Data Availability

The data and materials of the study can be obtained from the corresponding author upon request.

## Abbreviations

FBS: Fetal bovine serum
SP: Signal peptide
NOD: Non-obese diabetic
HE: Hematoxylin and eosin
BUN: Blood urea nitrogen
