# Supplementary material for: hUCMSCs carrying exenatide prevent T1DM by improving intestinal microflora composition and islet tissue damage repair
Source: Mol Med. 2022 Dec 13;28:155. doi: 10.1186/s10020-022-00526-0 (PMC9746121; doi:10.1186/s10020-022-00526-0)
Supplement: Supplementary file 2 — Additional file 2: Table S1. The serum levels of BUN, Scr and UAER as well as the number of podocytes in mice with different treatments at the 8th week. Table S2. Changes in the differential intestinal floras in the control and T1DM groups in the HMDAD database. Table S3. Correlation analysis between serum inflammatory cytokines and intestinal floras in T1DM mice. [file 10020_2022_526_MOESM2_ESM.docx]

**Additional file 2: Table S1** The serum levels of BUN, Scr and UAER as well as the number of podocytes in mice with different treatments at the 8^th^ week

| Group | n | BUN (mmol/L) | Scr (umol/L) | UAER (mg/24 hours) | podocytes number (/1000um2) |
| --- | --- | --- | --- | --- | --- |
| PBS | 12 | 8.82 ± 1.35 | 37.91 ± 3.07 | 65.58 ± 5.81 | 25.12 ± 2.68 |
| T1DM | 12 | 22.68 ± 2.09 | 124.03 ± 13.55 | 178.91 ± 19.78 | 6.67 ± 0.65 |
| Insulin | 12 | 16.03 ± 1.95ª | 81.27 ± 8.93ª | 136.05 ± 15.22ª | 12.42 ± 1.44ª |
| Ex-4 | 12 | 16.31 ± 1.81ab | 82.35 ± 8.05ab | 138.02 ± 10.27ab | 11.50 ± 1.38ab |
| hUCMSCs | 12 | 10.53 ± 0.99ab | 55.79 ± 6.25ab | 99.84 ± 8.63ab | 16.58 ± 1.68ab |
| hUCMSCs@Ex-4 | 12 | 7.89 ± 0.61abc | 40.03 ± 4.18abc | 70.55 ± 6.25abc | 21.25 ± 1.91abc |

Note: NOD, non-obese diabetic; T1DM, type 1 diabetes mellitus; hUCMSCs, human umbilical cord mesenchymal stem cells; BUN, blood urea nitrogen; Scr, serum creatinine; UAER, urinary albumin excretion rate. ^a^ *p* < 0.05 *vs.* NOD-T1DM mice. ^ab^ *p* < 0.05 *vs.* insulin. ^c^ *p* < 0.05 *vs.* hUCMSCs. Data comparisons among multiple groups were performed by one-way ANOVA.

**Additional file 2: Table S2** Changes in the differential intestinal floras in the control and T1DM groups in the HMDAD database

| Microbe name | Disease name | Evidence | PMID |
| --- | --- | --- | --- |
| *Bacteroides* | T1DM | Increase | 22043294 |
| *Dialister* | T1DM | Decrease | 22043294 |
| *Escherichia* | T1DM | Increase | 22043294 |
| *Eubacterium* | T1DM | Increase | 22043294 |
| *Haemophilus* | T1DM | Increase | 22043294 |
| *Phascolarctobacterium* | T1DM | Decrease | 22043294 |
| *Prevotella* | T1DM | Decrease | 22043294 |

Note: T1DM, Type 1 diabetes mellitus.

**Additional file 2: Table S3** Correlation analysis between serum inflammatory cytokines and intestinal floras in T1DM mice

|  | IL-1β | IL-6 | TNF-α | IL-10 | IL-13 | LPS |
| --- | --- | --- | --- | --- | --- | --- |
| *Escherichia* | 0.793 (*p* = 0.004) |  |  | -0.603 (*p* = 0.043) | -0.745 (*p* = 0.008) |  |
| *Phascolarctobacterium* | -0.696 (*p* = 0.014) | -0.700 (*p* = 0.014) |  |  | 0.791 (*p* = 0.003) |  |
| *Bacteroides* | 0.769 (*p* = 0.005) | 0.762 (*p* = 0.006) | 0.699 (*p* = 0.014) |  |  | 0.710 (*p* = 0.012) |
| *Haemophilus* | 0.859 (*p* = 0.001) |  |  |  |  | 0.730 (*p* = 0.009) |
| *Prevotella* | -0.608 (*p* = 0.040) | -0.692 (*p* = 0.016) | -0.867 (*p* = 0.001) | 0.608 (*p* = 0.040) | 0.720 (*p* = 0.011) |  |
| *Eubacterium* |  | 0.748 (*p* = 0.007) | 0.692 (*p* = 0.016) | -0.643 (*p* = 0.028) | -0.860 (*p* = 0.001) |  |
| *Dialister* |  | -0.828 (*p* = 0.001) |  | 0.597 (*p* = 0.044) | 0.614 (*p* = 0.037) |  |

Note: T1DM, type 1 diabetes mellitus; IL-, interleukin; TNF-α, tumor necrosis factor α; LPS, lipopolysaccharide. The correlation was reported by Spearman’s Rho (r), and the statistical significance was set as *p* < 0.05.
